# Experimental and Numerical Analysis of Bolted Repair for Composite Laminates with Delamination Damage

**DOI:** 10.3390/polym16202918

**Published:** 2024-10-17

**Authors:** Shan Xiao, Mingxuan Huang, Zhonghai Xu, Yusong Yang, Shanyi Du

**Affiliations:** 1 National Key Laboratory of Science and Technology on Advanced Composites in Special Environments, Harbin Institute of Technology, Harbin 150080, China; xiaoshanaaa@163.com (S.X.); sydu@hit.edu.cn (S.D.); 2 Shenyang Aircraft Design and Research Institute, Shenyang 110135, China; 3 No.1 Military Representative Office of Shenyang Military Representative Bureau of PLA Air Force Armament Department, Shenyang 110034, China; yitian753@sina.com

**Keywords:** composite, bolted repair, finite element method

## Abstract

Composite materials are widely used in aircraft due to the urgent need for high-quality structures in aerospace engineering. In order to verify the effectiveness of complex bolt repairs on composite structures, compression tests have been performed on three types (intact, damaged, and repaired) of composite plate specimens, and finite element simulation results of these three types’ specimens were obtained. The experimental results show that for damaged composite laminates, the strength recovery after bolt repair can reach an impressive 107%, and the delamination propagation caused by over-buckling deformation is considered to be the main cause of failure, which also suggests that although bolt repair can improve the strength of the specimens, it has a limited ability to inhibit delamination propagation. The simulation results of the finite element model in this paper are in good agreement with the actual experimental results, and the maximum error does not exceed 7.9%. In conclusion, this paper verifies the suitability of the proposed repair scheme in engineering applications and the correctness of the modeling method for repaired composite laminates.

## 1. Introduction

Carbon fiber-reinforced polymer composites (CFRPs) have been widely used in the aerospace field due to their excellent properties, including high specific strength, high specific modulus, and outstanding fatigue resistance. In today’s practical applications, they have gradually replaced traditional steel and aluminum materials and expanded into other fields such as automobiles, chemical processing equipment, and sports equipment that have high requirements for material performance, becoming a new generation of high-performance materials [1,2]. How to design, manufacture, and fully utilize the advantages of composite materials is a current research hotspot [3,4,5].

Research on the design of composite material repairs for aviation structures began in the 1970s [6,7], mainly originating from the repair of cracks in military aircraft used by the Air Force. Although the repair of composite materials was in its infancy at the time, it still provided a theoretical basis for the civilian repair of composite material structures. Airbus [8] and Boeing [9] both compiled corresponding repair manuals for reference and comparison, and specified the repair tolerances for civilian aircraft. According to the statistics of Turangel et al. [10], by the end of the 20th century, the number of repaired aircraft components had exceeded tens of thousands, indicating the necessity of research on composite material repair.

Currently, the common repair methods for composite materials mainly include three types: patch repair, scarf repair, and bolted repair. Among them, patch repair and scarf repair have been well developed. Mudassir Ali et al. [11] proposed that patch repair could be used as a temporary method to provide a solution for emergency repairs, but if critical components are damaged, the replacement or selection of other repair methods should be considered. Caliskan U et al. [12] verified the feasibility of using patch repair to restore the performance of impact-damaged laminates by studying the patch materials and patch thickness used in patch repair. Scarf repair has many commonly used design parameters, such as scarf angle, which have been extensively studied [13,14,15,16,17]. For example, Evren Sonat et al. [18] investigated the influence of different scarf overlap angles on joint failure modes and found that the 0-degree layer in the laminate often fails first, which means that stress concentration plays an important role in the failure behavior of the specimens. Hamza Bendemra et al. [19] studied the effect of joint parameters on the peak stress of adhesive lines for tapered and stepped scarf joints by comprehensively considering six factors, such as ply thickness, adhesive thickness, and taper angle. They found that stress concentration at the joint tip and step angle could be alleviated by introducing overlapping layers and properly changing the joint design parameters.

Bolted repair is a good way to restore the mechanical properties of composite materials. The specific operation process is usually to connect the composite patch or metal patch to the damaged part with fasteners to complete the repair [20,21,22]. This type of repair has a good anti-peeling performance, is less affected by the environment, allows disassembly and reassembly, can withstand large loads, and can be used for temporary repair in the field [23]. It can be said that bolted repair is a common means of repairing aircraft structures. However, most of the research in the literature focuses only on the repair of simple bolted connections in laminates. For example, Hu et al. [24] studied the bearing performance and failure mechanism of single bolt lap joints under different-temperature environments, Liu et al. [24] studied the mechanical properties of multiple bolt lap joints and conducted a finite element simulation to correspond to the experimental data, and Mehmet Caliskan [25] studied a simple laminated plate connected by four bolts. This is somewhat insufficient for practical engineering applications.

This paper first conducted compressive failure tests on delamination-damaged composite laminates repaired with complex bolts. By comparing the experimental results under different conditions of intact, damaged, and repaired specimens, the effectiveness of complex bolted repair on delamination-damaged composites was verified. The strength recovery rate of the damaged laminate after repair by this method can reach 107%. Subsequently, a high-precision finite element model was established based on the Hashin failure criterion and the progressive damage model (PDM) for simulation and comparative analysis, and the error was less than 7.9%. The numerical model and experimental verification demonstrated the effectiveness of this method for repairing delamination-damaged laminates.

## 2. Theoretical Background

### 2.1. Basic Formulations

First, we consider an ordinary composite laminate, and the displacement and stress fields can be expressed in the following form:

(1) $$U(x,y,z)=\left[\begin{array}{l} u(x,y,z)\\ v(x,y,z)\\ w(x,y,z) \end{array}\right]=\left[\begin{array}{l} u_{0}(x,y)+z\cdot \theta_{x}(x,y)\\ v_{0}(x,y)+z\cdot \theta_{y}(x,y)\\ w_{0}(x,y) \end{array}\right]$$

(2) $$\begin{array}{c} \varepsilon_{x}=\frac{\partial u_{0}}{\partial x}+z\cdot \frac{\partial \theta_{x}}{\partial x},\;\varepsilon_{y}=\frac{\partial v_{0}}{\partial y}+z\cdot \frac{\partial \theta_{y}}{\partial y}\\ \gamma_{xy}=\frac{\partial u_{0}}{\partial y}+\frac{\partial v_{0}}{\partial x}+z\cdot (\frac{\partial \theta_{x}}{\partial y}+\frac{\partial \theta_{y}}{\partial x}) \end{array}$$

$$\gamma_{xz}=\frac{\partial w}{\partial x}+\theta_{x},\;\gamma_{yz}=\frac{\partial w}{\partial y}+\theta_{y}$$

where $u_{0},v_{0},w_{0}$ represents the displacement, $\varepsilon_{x},\varepsilon_{y},\gamma_{xy}$ represents the strain, and z is variable in the z direction. The subsequent step involves deriving the resultant force calculation equation based on the general form of the constitutive equation:

(3) $$\left\{\begin{array}{c} N_{x}\\ N_{y}\\ N_{xy} \end{array}\right\}=\left[\begin{array}{ccc} A_{11} & A_{12} & A_{16}\\ A_{12} & A_{22} & A_{26}\\ A_{16} & A_{26} & A_{66} \end{array}\right]\left\{\begin{array}{c} \varepsilon_{x}^{0}\\ \varepsilon_{y}^{0}\\ \gamma_{xy}^{0} \end{array}\right\}+\left[\begin{array}{ccc} B_{11} & B_{12} & B_{16}\\ B_{12} & B_{22} & B_{26}\\ B_{16} & B_{26} & B_{66} \end{array}\right]\left\{\begin{array}{c} k_{x}^{0}\\ k_{y}^{0}\\ k_{xy}^{0} \end{array}\right\}$$

(4) $$\left\{\begin{array}{c} M_{x}\\ M_{y}\\ M_{xy} \end{array}\right\}=\left[\begin{array}{ccc} B_{11} & B_{12} & B_{16}\\ B_{12} & B_{22} & B_{26}\\ B_{16} & B_{26} & B_{66} \end{array}\right]\left\{\begin{array}{c} \varepsilon_{x}^{0}\\ \varepsilon_{y}^{0}\\ \gamma_{xy}^{0} \end{array}\right\}+\left[\begin{array}{ccc} D_{11} & D_{12} & D_{16}\\ D_{12} & D_{22} & D_{26}\\ D_{16} & D_{26} & D_{66} \end{array}\right]\left\{\begin{array}{c} k_{x}^{0}\\ k_{y}^{0}\\ k_{xy}^{0} \end{array}\right\}$$

where $k_{x}^{0},k_{y}^{0}$ and $k_{xy}^{0}$ represents curvature, $N_{x},N_{y},N_{xy}$ and $M_{x},M_{y},M_{xy}$ represent the internal force and moment of the laminate, respectively, and $A_{ij},B_{ij},D_{ij}$ represent the in-plane stiffness, coupling stiffness, and bending stiffness, respectively.

By Equations (1)–(4) mentioned above, the stress–strain relationship of the finite element method (FEM) can be established, and the finite element model can be calculated by solving the matrices, to obtain some reliable numerical solutions. In this paper, the FEM is used for damage and limit load analysis.

### 2.2. Progressive Damage Analysis

The strength prediction method employed in this study is the progressive damage analysis approach based on energy dissipation degradation. During computation, material failure initiation is determined using the two-dimensional Hashin failure criterion [26]. For unidirectional plates, the shear constitutive relation follows the subsequent expression:

(5) $$\gamma_{12}=(\frac{1}{G_{12}^{0}})\tau_{12}+\alpha \tau_{12}^{3}$$

The Hashin failure criterion encompasses five distinct modes of failure, namely matrix tensile failure, matrix compression failure, fiber tensile failure, fiber compression failure, and fiber-matrix shear failure. The linearized form of this criterion is as follows:

Matrix tensile failure

(6) $$e_{m}^{2}={\left(\frac{\sigma_{2}}{Y_{t}}\right)}^{2}+{\left(\frac{\tau_{12}}{S_{C}}\right)}^{2}$$

Matrix compression failure

(7) $$e_{m}^{2}={\left(\frac{\sigma_{2}}{Y_{C}}\right)}^{2}+{\left(\frac{\tau_{12}}{S_{C}}\right)}^{2}$$

Fiber tensile failure

(8) $$e_{m}^{2}={\left(\frac{\sigma_{1}}{X_{t}}\right)}^{2}+{\left(\frac{\tau_{12}}{S_{C}}\right)}^{2}$$

Fiber compression failure

(9) $$e_{m}^{2}={\left(\frac{\sigma_{1}}{X_{C}}\right)}^{2}$$

Shear failure of fiber matrix

(10) $$e_{m}^{2}=(\frac{\sigma_{1}}{X_{C}}{)}^{2}+{\left(\frac{\tau_{12}}{S_{C}}\right)}^{2}$$

**Figure 1:**
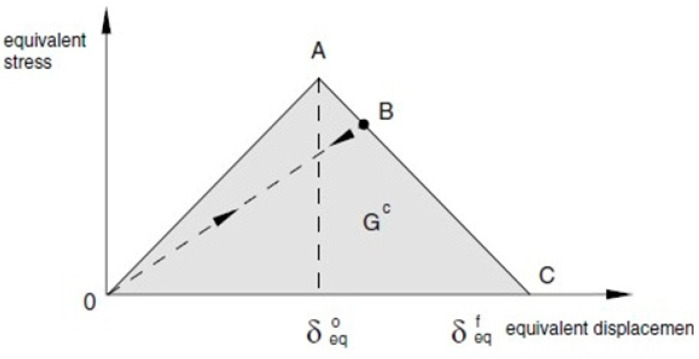
Linear degradation model used in Abaqus.

Next, the material stiffness was subsequently degraded by manipulating the internal damage variables based on the corresponding energy release rate of each delamination, aiming to simulate the progressive damage behavior of the material and conduct a comprehensive analysis of structural failure. Linear degradation, as illustrated in Figure 1, was employed for this purpose.

To ensure the stability of the calculations, such models often require the use of viscous regularization methods. In implicit analysis, increasing the viscosity in the degradation model can improve the convergence of the model, but it can also affect the rate of stiffness degradation after composite material failure. In explicit analysis, the viscosity parameter in the degradation model can be removed because convergence issues do not exist. However, for specimens with buckling deformation, the time step needs to be long enough to accurately capture the stiffness reduction caused by buckling. As for compression load simulations, implicit analysis methods are used due to their unconditional stability. Therefore, the time increment Δt is relatively larger compared to the explicit analysis method. For nonlinear problems, several iterations are required to obtain a solution that satisfies a given tolerance for each typical incremental step. Each Newton iteration provides a correction value for the displacement increment. The instantaneous equation to be solved at each iteration is

(11) $${\hat{K}}_{j}c_{j}=P_{j}-I_{j}-M_{j}{\ddot{u}}_{j}$$

where ${\hat{K}}_{j}$ is the linear combination of tangential stiffness matrix and mass matrix of this iteration.

## 3. Experiment and Simulation Preparation

### 3.1. Specimen Description

This paper includes three types of test specimens: non-destructive, damaged, and repaired. These specimens are fabricated from T300/QY8911 carbon fiber/epoxy unidirectional thin plates with a fiber volume fraction of 60% and a single-layer thickness of 0.12 mm. The specific material properties can be found in Table 1. The composite specimen laminates are laid up [±45/0/45/90/−45/0/−45/0/45/45/90/−45/0]s, consisting of 28 layers with a total thickness of 3.36 mm. All composite laminates in this paper were cured in an autoclave using standard autoclave procedures to complete the curing, and vacuum bags were used to provide the required pressure during curing, Teflon film is used to prefabricate the delamination. Once cured, the laminates were cut into experimental standard shapes using a chainsaw cutter, and for damaged specimens, patches and laminates are bolted together for repair. Prior to delivery, all specimens undergo manufacturing compliance checks and quality testing using ultrasonic reflection. The test results indicated that no non-conformities are detected except for the pre-existing defects.

**Figure 2:**
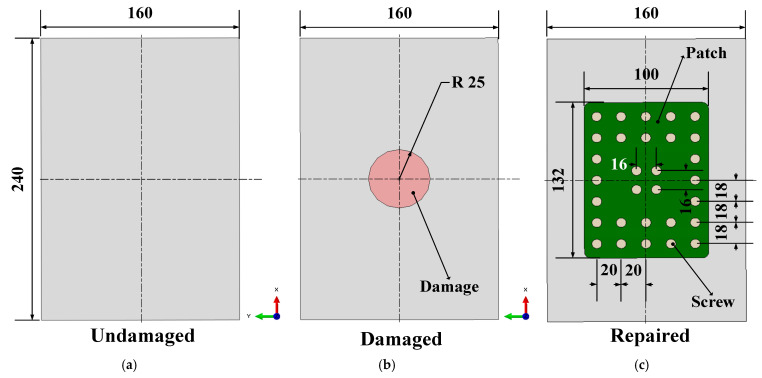
Composite laminates with intact (**a**) and pre-existing delamination damage (**b**) and repaired with bolts (**c**).

The ability of bolted repair to repair composite plates is verified through compression tests on fabricated composite material specimens. The specific dimensions of the initial laminates are shown in Figure 2a. The specific parameters of the composite laminates with prefabricated delamination damage are shown in Figure 2b. The delamination damage is prefabricated with polytetrafluoroethylene film.

**Figure 3:**
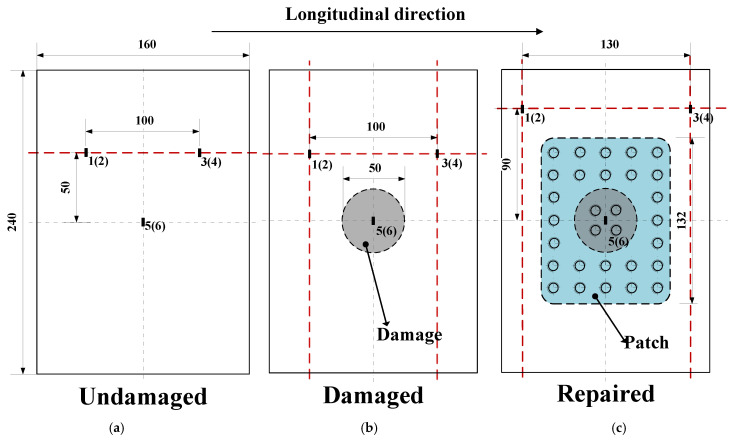
Distribution situation and numbers of strain gauge: intact (**a**) and pre-existing delamination (**b**) and repaired with bolted joint (**c**).

**Figure 4:**
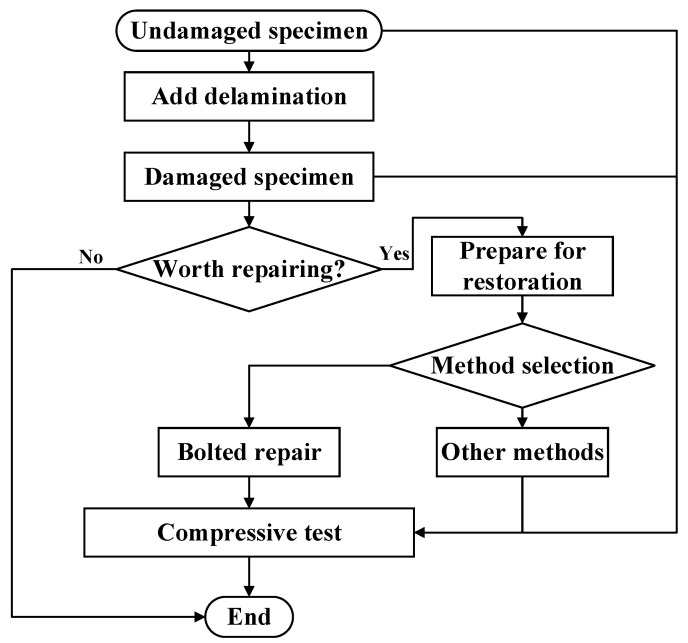
Repair operation flowchart.

As shown in Figure 2b, the prefabricated damage represents 5.11% of the total laminate, and in actual service, damage of this size can already lead to the performance degradation of the whole structure. According to our experience, the length of the short side of the metal plate used for repair is 1.5–2.5 times the diameter of the damaged area, and in this paper, we chose the length of the short side to be 2 times the diameter of the damaged area in order to ensure the repair effect. For the pitch of the bolts, it should be more than 2 times the bolt diameter and less than 6 times the bolt diameter, and in this paper, the pitch is between 16 and 20 mm, which is a reasonable range based on experience. The repair test specimens have specific dimensions as shown in Figure 2c, using mechanical repair fasteners (product code Q/1S113-2008) made of titanium alloy TC4. The adhesive used is J-296, and the metal patch used for the laminate reinforcement is made of 7050-T7451 aluminum alloy. The position of the experimental adhesive strain gauge is shown in Figure 3, and the specific experimental procedure is shown in Figure 4. First, we made undamaged laminates, and then by adding delamination, we made damaged laminates. Afterwards, we obtained all three laminate specimens by determining whether the damaged laminates were worth repairing and then performing the specific repair method. Finally, by placing strain gauges as shown in Figure 3 and performing compression tests, we verified the effectiveness of bolt repairs on the composites.

### 3.2. Experimental Setup

**Figure 5:**
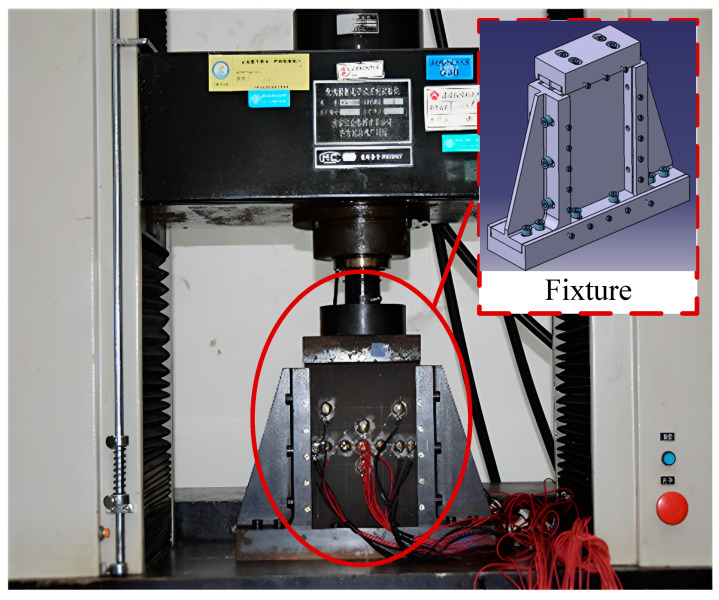
Experimental setup.

The compression tests are divided into three groups: intact specimens (WSC), damaged specimens (YSC), and repaired specimens (XLC). To ensure the generality of the experimental data, each group of experiments included three specimens numbered 1, 2, and 3. Before the experiment, we assumed that the complex damage could be simplified to a circle, with the radius being the distance of the damage boundary from the two most distant points. For this reason, circular delamination has been used in this paper to prefabricate the damaged laminate. The experimental equipment used in this study is a WDW-E200D microcomputer-controlled electronic universal testing machine (Jinan Shijin Group Co., Ltd., Jinan, China) with a capacity of 25 tons, as shown in Figure 5. The device consists of an upper clamping platform, a slide rail, a lower supporting platform, and a set of pressure sensing modules. During the static compression test, the load and displacement measurements are recorded and outputted by the built-in sensors of the testing machine. Photographs and videos are captured by a Nikon D5300 camera (Nikon Corporation, Jinan, China).

The strain measurements are obtained using a Jiangming JM3813 (Jingming Technology Co., Ltd., Yangzhou, China)static strain testing and analysis system from Yangzhou, which has a measurement accuracy of ±2 με, is calibrated and qualified for use within its valid period. The strain gauges used are the BE120-3AA resistive strain gauges from AVIC Electric Measurement Technology Co., Ltd., Hanzhong City, China, with a resistance value of 120.0 ± 0.1 Ω and a sensitivity coefficient of 2.22 ± 0.1%. During the tests, a 1/4 bridge connection is used, and compensation strain gauges are utilized to eliminate the effect of the ambient temperature. Before loading, the specimens must be carefully aligned with the neutral axis. During loading, the lower end of the specimen remains stationary, while the upper end is subjected to a compressive load.

### 3.3. Finite Element Model

**Figure 6:**
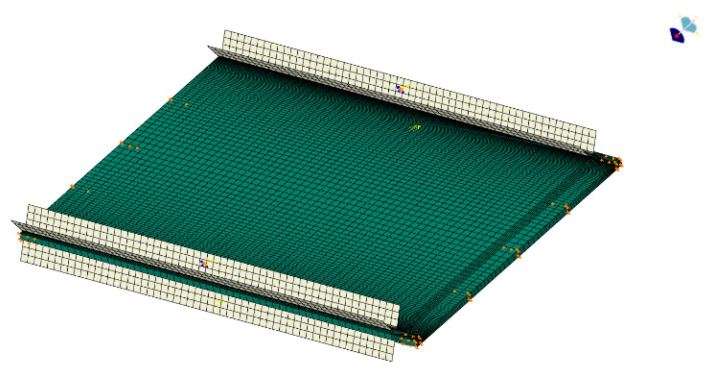
Finite element model for initial composite laminates, where green is the laminate model and white is the fixture model.

In order to accurately simulate the compression test, the outer displacements of the contact surfaces between the top and bottom of the test piece and the fixtures (U3 = 0) are constrained, and the side fixtures are modeled as discrete rigid bodies and fixed on both sides of the test piece, as shown in Figure 6. Contact between the test piece and the fixtures is established, and the mesh in the contact area is refined to ensure convergence.

However, since the buckling analysis does not support contact algorithms, the fixtures are removed, and the initial contact of the test piece with the fixtures is constrained on the four edges with outer displacement (U3 = 0). The material parameters used in the numerical simulation process are shown in Table 1. This prepreg is made from T300 carbon fiber and QY8911 epoxy resin (Guangwei Composites Co., Weihai, China).

**Figure 7:**
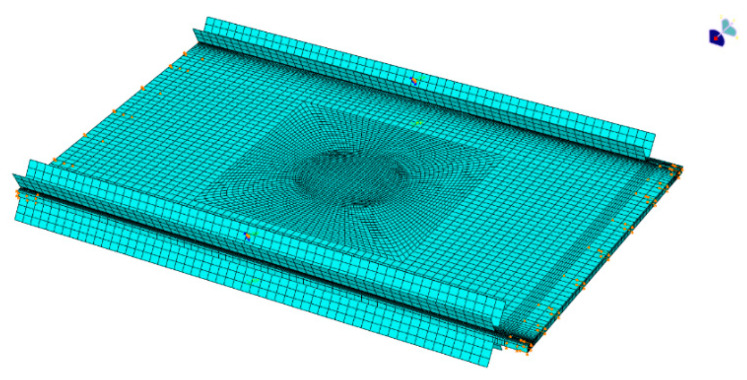
Finite element model for composite laminates with delamination.

The delamination of the damaged laminate is circular and located between the 8th and 9th layers, with a diameter of 50 mm. Assuming a delamination interface thickness of 0.02 mm, the measured interlaminar fracture toughness values for Mode I and Mode II in T300/QY8911 are determined to be 0.252 mJ·mm^−2^ and 0.665 mJ·mm^−2^, respectively. According to the literature [27,28], the cohesive element stiffness used for simulating delamination is set at 10^6^, while the strength value is taken as 60% of the matrix tensile strength. The length of the cohesive zone is determined to be 2 mm based on the Hillerborg model, with at least three meshes arranged within this region, as suggested by relevant studies. The final model configuration can be observed in Figure 7.

The repaired finite element model does not exhibit asymmetric buckling under compressive loading. To enhance computational efficiency, only half of the specimen is modeled for finite element analysis. The friction coefficients between bolts and patches, bolts, and laminates, as well as patches and laminates are set to 0.1 based on actual conditions. A gap of 0.05 mm is introduced between the bolt hole and laminate.

**Figure 8:**
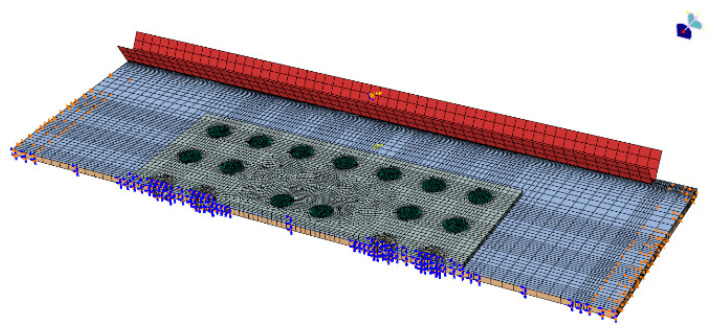
Finite element model of repaired delaminated composite laminates.

For the standard compression test fixture used in this paper, the upper side of the simulation model should be loaded in compression (U1 = 3) and the lower side of the model was completely fixed (U1 = U2 = U3 = UR1 = UR2 = UR3 = 0). Since only half of the model was built for computational efficiency, the symmetry boundary condition should be imposed on its symmetry plane (U2 = UR1 = UR3 = 0). In addition, in order to match the experimental boundary conditions as closely as possible, we built fully fixed rigid body fixtures on the left and right sides of the laminate to simulate the simply supported boundary conditions at the edges of the laminate. The mesh and boundary conditions of the model are illustrated in Figure 8.

## 4. Results and Discussion

### 4.1. Comparative Analysis of Limit Load

#### 4.1.1. Intact Composite Laminate

**Figure 9:**
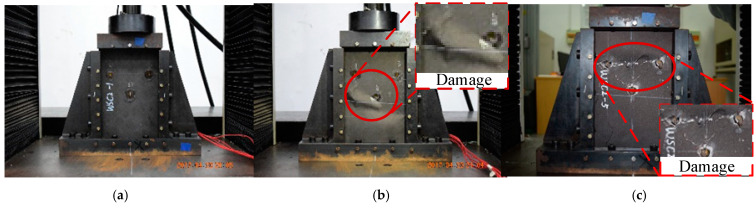
Intact composite laminates after compression test ((**a**–**c**) correspond to specimens 1, 2, and 3, respectively).

The compression failure test is first performed on the undamaged laminated composite plate to measure its maximum strength. The failure images after the test are shown in Figure 9.

During the experiment, it is observed that the failure position of each specimen is different, but the failure modes are similar. The central position of the specimen was first crushed, followed by delamination and fiber fracture, which propagated through the entire thickness and then rapidly spread to both sides, resulting in the entire specimen breaking. The failure positions observed in the experiment are all in the center of the specimen, which is considered a good and acceptable failure type.

**Figure 10:**
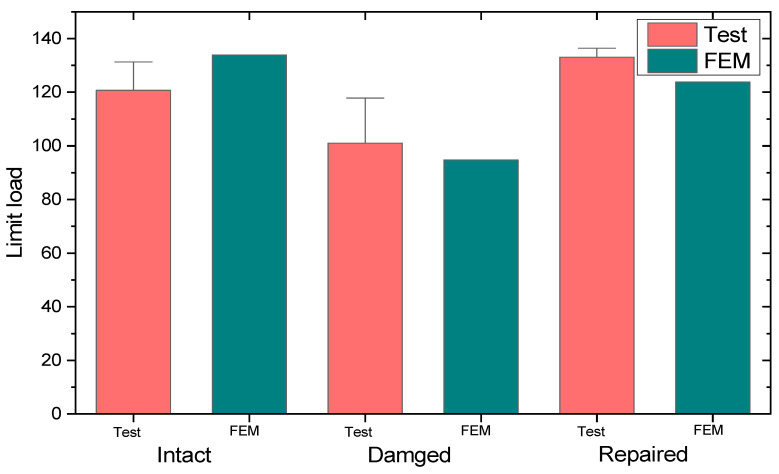
Comparison of experimental and simulation results.

At a displacement of 1.807 mm the specimen fails with a strength of 133.907 kN. The average strength measured during the test is 124.038 kN, resulting in an error of 7.9% compared to the FEM results. The failure behavior of the specimen is similar to the simulated results, indicating a relatively accurate analysis. All of the experimental and simulation results in this paper are compared in Figure 10. For intact specimens, the results of the experiment and simulation are shown in Table 2.

#### 4.1.2. Damaged Composite Laminate

**Figure 11:**
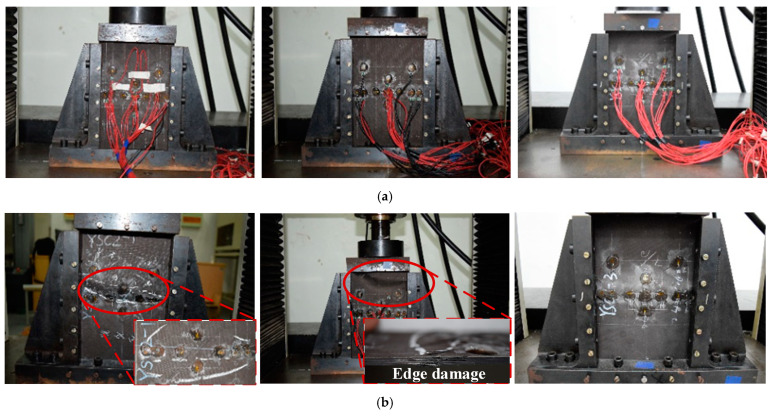
Damaged composite laminates before (**a**) and after (**b**) compression test.

Compression tests are then carried out on laminates with delamination damage. The resulting failures are shown in Figure 11. It is clear that the presence of delamination damage leads to earlier crack initiation. The damaged specimens exhibit a typical failure mode accompanied by sublayer failure. This eventually leads to severe damage and early failure due to excessive damage accumulation. In addition, Figure 11 provides many magnified images of the damage locations, through which the damage mechanism can be observed [29]. It can be seen that minor damage such as matrix cracks are not able to cause an overall destruction of the laminate, and the breakage and pulling out of fiber always means that the laminate is severely damaged. As a result, the compression failure experiment carried out on laminates with embedded delamination not only provides an insight into the actual failure mode, but also shows that delamination damage degrades the mechanical performance of laminates and thus leads to sudden failure.

The limit load obtained during the experiment and FEM analysis are shown in Figure 10. Specimens WSC1 and WSC3 exhibit sub-layer buckling due to pre-existing delamination, characterized by a buckling mode consisting of two half-waves. In contrast, specimen WSC2 exhibits a failure mode similar to the undamaged specimens, possibly because no mode transformation occurs and the buckling mode consists of only one half-wave, which remains unaffected by delamination damage throughout the structure. It can be concluded that the occurrence of buckling accelerates damage propagation, leading to premature failure of the laminates. In Table 3, the average measured intensity is 100.972 kN, which gives an error of 6.2% from the simulated results, indicating a high degree of agreement between the simulated and experimental results.

#### 4.1.3. Repaired Composite Laminate

**Figure 12:**
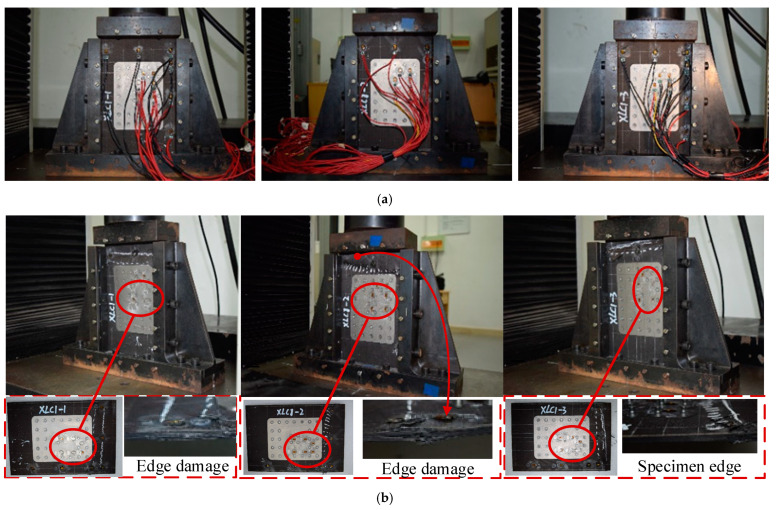
Images of the repaired composite laminates before (**a**) and after (**b**) compression test.

After bolted repair, compression damage tests are carried out on the composite laminates. In general, the repaired laminates exhibit a higher strength compared to their damaged and initial states. Typical failure modes are shown in Figure 12.

During the experiment, the XLC1 specimen exhibited a two half-wave buckling mode, with the repair patch strategically positioned at both the peak and trough of the wave. When the buckling load was reached, excessive bending deformation occurred at the edge of the repair patch, ultimately leading to the failure of the specimen. A similar failure mode was observed for specimens XLC2 and XLC3, with one side experiencing initial crushing at the top of the side fixture, followed by a rapid increase in bending deformation over the entire cross section, leading to subsequent buckling failure on the opposite side.

The experimental and simulation results are shown in Figure 10 and indicate that the specimens have similar buckling patterns. However, since the damage propagation bypassed the bolt area, the damage required a higher energy, making it more difficult to fail the whole material. Looking at the whole set of specimens, the optimum strength recovery among them was as high as 107%, indicating that bolt repair is an effective method of repairing damaged composites.

In Table 4, the experimental average strength is measured to be 133.069 kN, with a finite element analysis error of 7.45%. At a displacement of 0.623 mm, the test piece loses its bearing capacity and reaches a maximum load of 123.842 kN. The finite element simulation accurately captures the occurrence of defects within the test piece during actual testing, leading to an overall modulus decrease and simulating the process by which the test piece undergoes compression-induced buckling failure. However, due to the use of a relatively simple damage evolution model, the damage occurred relatively late, but the results are not significantly different. This is because an engineering-appropriate calculation model is used, and the accuracy of the model is improved. It can be said that the error of the finite element analysis is maintained within ±10%, and the simulation results are quite accurate.

### 4.2. Comparative Analysis of Strain

**Figure 13:**
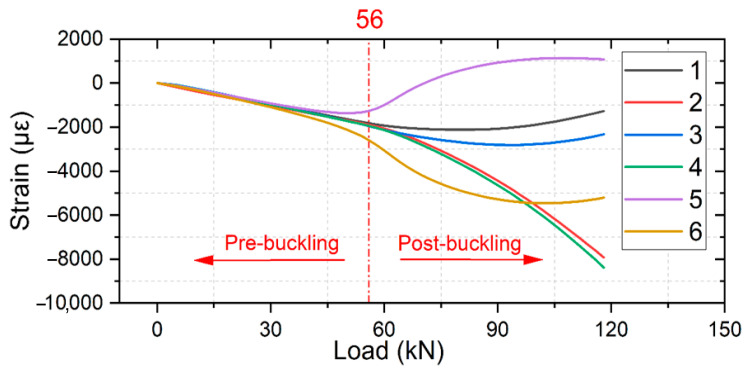
Strain response of initial composite laminates.

**Figure 14:**
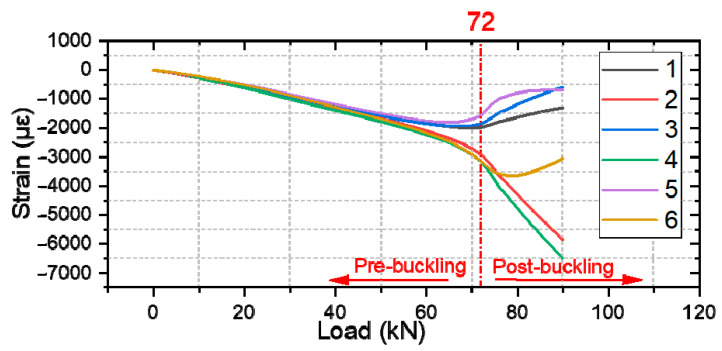
Strain response of damaged composite laminates.

**Figure 15:**
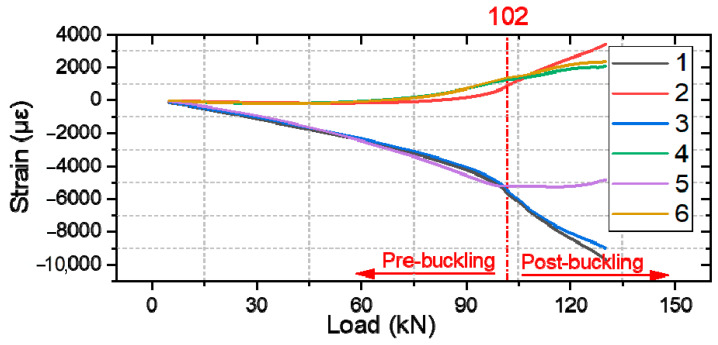
Strain response of repaired composite laminate.

In this section, we compare the strain measurements of different types of specimens, where the strain of specimen #1 of each group is measured using strain gauges. Figure 13, Figure 14 and Figure 15 presents strain-load curves illustrating the strain variation at the measurement point.

For the initial composite laminates (Figure 13), when the load pressure reaches approximately 56 kN, all three specimens show divergent trends in their strain curves, indicating the onset of buckling deformation and resulting in uneven deformations in different directions due to laminate bending. This leads to uneven stress distribution and delamination propagation within the laminates. In the simulation, this specimen buckled at a displacement of 0.615 mm with a corresponding buckling load of 75.326 kN. The mean buckling load obtained from the experiment is 60.667 kN, resulting in an analysis error of 24.2%.

For damaged composite laminates (Figure 14), when the specimen buckled, it initially exhibited a half-wave buckling mode. The laminate then undergoes a mode transition as the delamination expands, resulting in a slight reduction in load. Finally, the delamination rapidly expands towards the edges causing the laminate to fail. During this process, the flexural deformation of the specimen continues to increase with constant fiber breakage and occasional load reduction. This continues until a certain load value is reached, at which point the structure breaks and the load carrying capacity is lost.

For repaired composite laminates (Figure 15), it is difficult to plot the strain gauges at the same position as in Figure 13 and Figure 14, so the two measurement points 1(2) and 3(4) are moved, and the measured data after moving can still show the damage process of the whole specimen very well. Significant strain changes occur when loaded to approximately 102 kN. The positive increase in strain readings at these positions indicates the delamination of the repaired laminates at the crest and trough locations. Failure is expected to occur near the edges of the specimen where significant deformation and shear damage is likely to occur.

### 4.3. Failure Analysis

#### 4.3.1. Intact Composite Laminate

**Figure 16:**
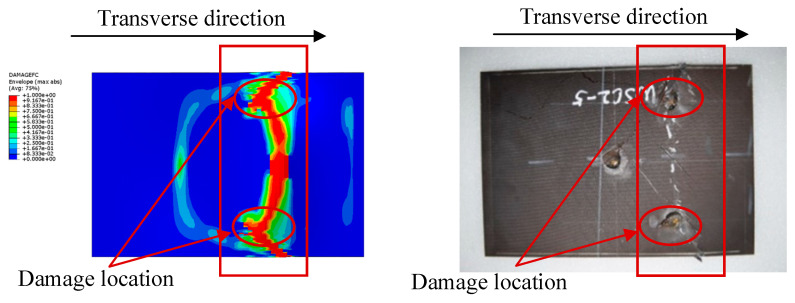
Fiber damage cloud map and experimental result graph.

The damage modes of both WSC2 and WSC3 tested were center failure, whereas the damage mode of WSC1 was end crush. The end failure of the specimen may be due to the excessive initial deflection of the specimen. The finite element analysis shows that the failure mode is consistent with WSC3, i.e., two half-wave buckling. A comparison of the FEA and test results is shown in Figure 16. The damage occurred first due to the higher force at the symmetric center axis. It can also be observed that damage occurred near the edges of the undamaged specimen to which the load was applied, which indicates that delamination or matrix damage may also occur at the edges of the specimen during actual testing. Subsequently, as the damaged portion loses its load-bearing capacity, the load on the remaining portion increases, leading to damage extending from the center of the specimen to the sides, and ultimately leading to a complete loss of load-bearing capacity of the entire specimen. The test results are in good agreement with the simulation results.

#### 4.3.2. Damaged Composite Laminate

**Figure 17:**
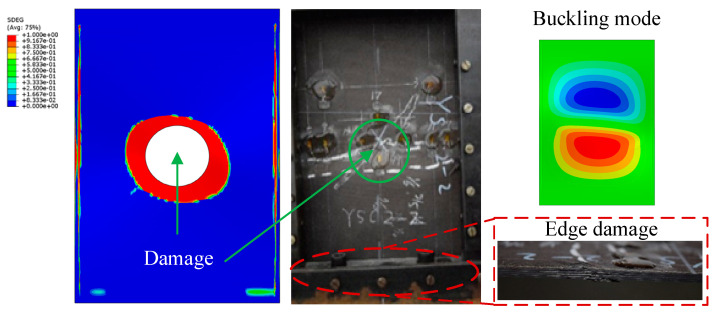
Predicted displacement contour map and experimental result.

The experimental results show that YSC1 and YSC3 failed due to delamination propagation, and the failure mode of YSC2 in Figure 11 is different from the other two specimens, and such a failure mode comes from the stress concentration at the end. Referring to ASTM D6641/D6641M, it can be seen that such damage mode is unacceptable [30]. The finite element analysis reveals that the failure mode of the specimens was delamination propagation, which is consistent with the experimental results. The delamination interface damage distribution at the ultimate load is shown in Figure 17, where the damage was initiated at the end of the delamination and quickly propagated towards both sides with the application of compressive load. This indicates that in the repair process of composite delamination, the density of screws should be increased on both sides to control the propagation of delamination and to transfer the main load to the patch through good fixation to prevent the adverse effects of delamination on the performance of the laminated plate. The deformation during failure predicted by the finite element analysis is shown in Figure 17, and the simulation results are in good agreement with the experimental results.

#### 4.3.3. Repaired Composite Laminate

**Figure 18:**
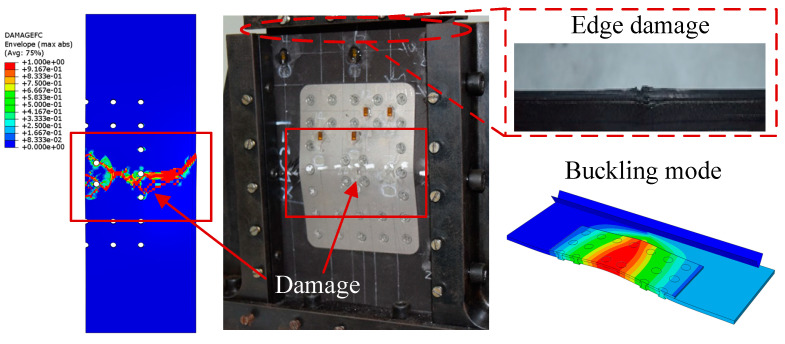
Calculation results of repaired compression test specimen.

During the compression process, the edge structure stiffness of the patch suddenly decreases, and its bending resistance ability also decreases, resulting in a significant bending deformation. The deformation after the model bearing is shown in Figure 18, where the peak and valley of the buckling deformation occur at both ends of the patch, which is consistent with the experimental results. It is speculated that the presence of screws constrained the deformation of the specimen, making the two ends of the patch more susceptible to stress concentration, resulting in a large amount of buckling deformation and ultimately causing failure due to the compression damage of the composite plate fibers at the edge of the patch. Although drilling composite laminates can easily cause delamination damage, this situation does not occur in the experiment, so there is no need to consider the damage caused by drilling during the simulation process. The failure modes obtained by the finite element analysis and experiment are shown in Figure 18, and the two are in good agreement.

## 5. Conclusions

In this paper, the reliability of composite laminates repaired with bolts is verified by experiment and simulation, and an engineer-friendly FEM model for composite repair has been proposed. By comparing the experimental and simulation results on undamaged, damaged, and repaired composite laminates, the error of this model is small compared to the experiment. The main conclusions are as follows:

1. In the static compression tests of composite laminates, the pre-existing single delamination can cause an 18.4% decrease in strength. After bolt repair, 107% strength recovery is achieved, indicating that the repair is effective.
2. The damage mechanism of composite laminates is revealed. Delamination in both intact and damaged laminates can propagate freely, and in damaged laminates, it is easier due to the presence of prefabricated delamination. In bolt-repaired laminates, the damage propagation must bypass the bolt, which changes the damage mechanism and allows them to carry greater loads.
3. The results of the finite element analysis show that the maximum prediction error for the strength of all specimens is not more than 7.9%. In addition, the failure modes and locations predicted by the finite element model are consistent with the experimental results. These results show that the modeling method used in this paper is reliable.

## Figures and Tables

**Table 1:** Mechanical parameters of T300/QY8911 unidirectional lamina.

Parameter	Symbol	Value	Unit
Longitudinal elastic modulus	*E*_1_	135	GPa
Transverse and normal elastic modulus	*E*_2_, *E*_3_	8.8	GPa
Poisson’s ratio	*ν*_12_	0.33	-
Shear modulus	*G*_12_, *G*_13_, *G*_23_	4.47	GPa
Longitudinal tensile strength	*X*_T_	1548	MPa
Longitudinal compressive strength	*X*_C_	1226	MPa
Transverse tensile strength	*Y*_T_	55.5	MPa
Transverse compressive strength	*Y*_C_	110.5	MPa
Shear strength	*S*_12_, *S*_23_	89.9	MPa

**Table 2:** Bearing capacity of intact composite laminates.

Specimen	Test Results/kN				FEM Results/kN	Error
	Buckling Load	Average	Limit Load	Average		
WSC1	56	60.7	118.50	124.04	133.91	7.9%
WSC2	54		122.32			
WSC3	72		131.30			

**Table 3:** Bearing capacity of damaged composite laminates with delamination.

Specimen	Test Results/kN				FEM Results/kN	Error
	Buckling Load	Average	Limit Load	Average		
YSC1	72	68.7	90.70	100.97	94.75	6.2%
YSC2	68		117.82			
YSC3	66		94.39			

**Table 4:** Bearing capacity of repaired composite laminates.

Specimen	Test Results/kN				FEM Results/kN	Error
	Buckling Load	Average	Limit Load	Average		
XLC1	102	109.3	131.13	133.07	123.84	7.45%
XLC2	114		136.41			
XLC3	112		131.67			

## Data Availability

Data are contained within the article.
